# Radiologic-Pathologic Correlation: Is There an Association Between Contrast-Enhanced Mammography Imaging Features and Molecular Subtypes of Breast Cancer?

**DOI:** 10.7759/cureus.64791

**Published:** 2024-07-18

**Authors:** Beatrice Wing-Tung Cheng, Tsz Yan Ko, Yee Tak Alta Lai

**Affiliations:** 1 Department of Radiology, Pamela Youde Nethersole Eastern Hospital, Hong Kong, HKG

**Keywords:** contrast-enhanced mammogram, basal-like, her2, luminal, breast cancer subtypes, radiologic-pathologic correlation, breast cancer

## Abstract

Objective: This study aims to assess the correlation between imaging features of contrast-enhanced mammography (CEM) and molecular subtypes of breast cancer.

Methods: This is a retrospective single-institution study of patients who underwent CEM from December 2019 to August 2023. Each patient had at least one histologically proven invasive breast cancer with a core biopsy performed. Patients with a history of breast cancer treatment and lesions not entirely included in the CEM images were excluded. The images were interpreted using the American College of Radiology Breast Imaging Reporting and Data System (ACR BI-RADS) lexicon for CEM, published in 2022. Different imaging features, including the presence of calcifications, architectural distortion, non-mass enhancement, mass morphology, internal enhancement pattern, the extent of enhancement, and lesion conspicuity, were analyzed. The molecular subtypes were studied as dichotomous variables, including luminal A, luminal B, HER2, and basal-like. The association between the imaging features and molecular subtypes was analyzed with a Fisher's exact test. Statistical significance was assumed when the p-value was <0.05.

Results: A total of 31 patients with 36 malignant lesions were included in this study. Sixteen lesions (44.4%) were luminal A, four lesions (11.1%) were luminal B, 10 lesions (27.8%) were HER2, and six (16.7%) were basal-like subtypes. The presence of calcifications was associated with the HER2 subtype (p=0.024). Rim-enhancement on recombined images was associated with a basal-like subtype (p=0.001). Heterogeneous enhancement on recombined images was associated with non-basal-like breast cancer (p=0.027). No statistically significant correlation was found between other analyzed CEM imaging features and molecular subtypes.

Conclusion: CEM imaging features, including the presence of calcifications and certain internal enhancement patterns, were correlated with distinguishing breast cancer molecular subtypes and thus may further expand the role of CEM.

## Introduction

Breast cancer is the most frequently diagnosed cancer worldwide [1]. It is a highly heterogenous neoplasm with a variety of histologic patterns, which can be classified into molecular subtypes: luminal-like, human epidermal growth factor receptor 2 (HER2)-enriched, and basal-like [2]. The molecular subtypes are categorized based on tumor biomarkers’ expression status: estrogen receptor (ER), progesterone receptor (PR), and HER2/neu overexpression. The molecular subtypes have important differences in incidence, disease progression, survival, and response to treatment. They also play critical roles in treatment planning and disease prognosis.

A luminal-like tumor is the most common type of breast cancer (60-70% of all tumors) and is divided into two subgroups: A and B. The luminal A tumor has the best prognosis, while the luminal B subtype has poorer clinical outcomes [2]. Hormone therapy is part of the luminal-like tumor treatment regimen. HER2-enriched is a less common subtype, accounting for 12-20% of all breast cancer [2]. HER2-directed therapy improves the clinical outcomes of HER2-positive subtype breast cancer. Basal-like tumors comprise about 15% of all invasive breast cancer, which is often high-grade at the time of diagnosis and has a high rate of local and distant recurrence [3].

Contrast-enhanced mammography (CEM) is a diagnostic imaging modality that uses a dual-energy technique and iodinated contrast media to provide images that display morphological and anatomical features of breast tumors as well as reflect tumor neovascularity, similar to breast MRI [4]. According to previous studies, CEM has non-inferior diagnostic performance compared to breast MRI [5,6]. It is also more convenient and has a lower cost compared to an MRI. Some studies have shown correlations between the imaging features of breast MRI and molecular subtypes [7,8]. As a result, we hypothesized that imaging features of CEM may also have correlations with molecular subtypes.

The aim of this study was to describe and discuss the association between CEM imaging features and different molecular subtypes of breast cancer. Accordingly, we investigated the imaging features of molecular subtypes in CEM and compared them with the pathological results. This may provide important imaging information for specialized treatment planning and prognosis evaluation.

This article was previously presented as a meeting abstract at the 22nd Asian Oceanian Congress of Radiology on March 24, 2024.

## Materials and methods

This is a retrospective single-institution study conducted in a tertiary regional hospital. Approval from the Hospital Authority Central Institutional Review Board was obtained (approval number: CIRB-2024-033-4), and individual consent for this retrospective analysis was waived.

Patients

**Figure 1:**
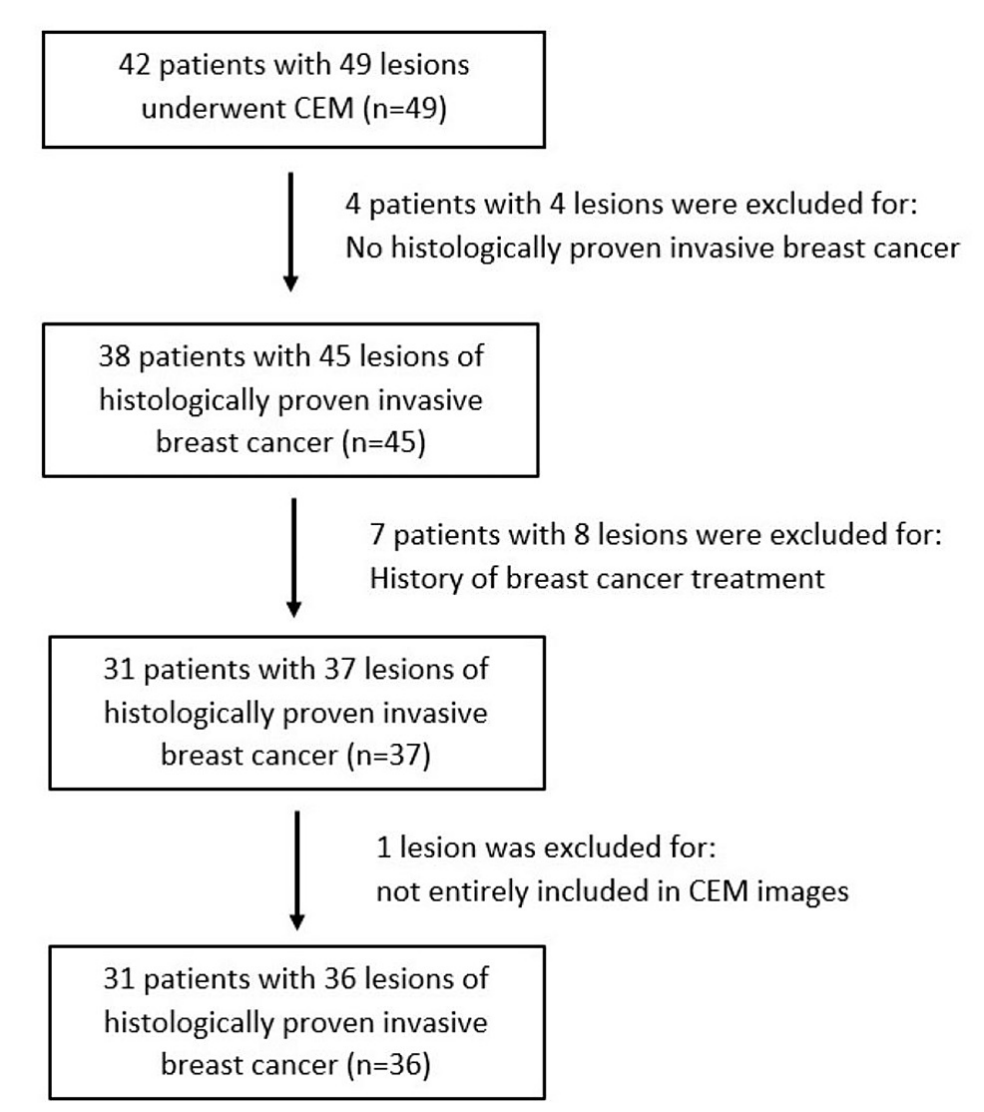
Patient inclusion and exclusion workflow CEM: contrast-enhanced mammography

A retrospective review of departmental cases with CEM performed from December 2019 to August 2023 retrieved from RIS was conducted. Inclusion criteria included female patients aged more than 18 years old who had at least one invasive breast cancer diagnosed based on core needle biopsy and immunohistochemistry, with CEM done in the institute. Exclusion criteria included patients with a history of breast cancer treatment (e.g., operation, radiotherapy, chemotherapy, or hormonal treatment) and lesions not entirely included in the CEM images (Figure 1).

CEM examination

**Figure 2:**
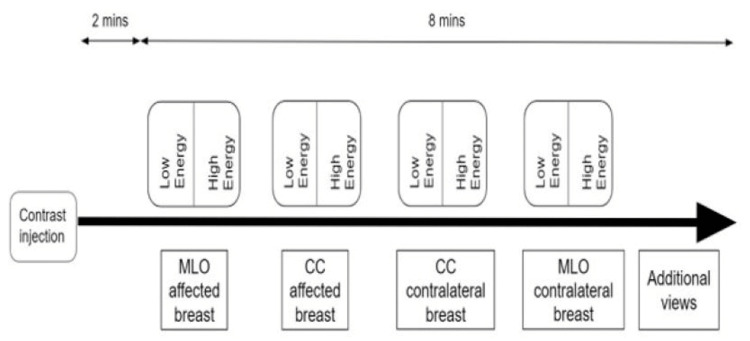
CEM workflow CEM: contrast-enhanced mammography, CC: craniocaudal, MLO: mediolateral oblique

A CEM examination was performed utilizing the Hologic Selenia Dimensions Mammography System (Hologic, Inc., Marlborough, MA, USA). The CEM contrast agent used was Omnipaque^TM^ (iohexol, GE Healthcare, Chicago, IL, USA). The volume of contrast administered would be 1.5 ml/kg of the patient’s body weight at a rate of 3 ml/sec through a power injector. CEM images were then acquired two minutes after contrast injection and completed within ten minutes. CEM low- and high-energy images in standard craniocaudal (CC) and mediolateral oblique (MLO) projections of each breast were obtained (Figure 2). CEM high-energy images were used in post-processing to produce the recombined images that showed areas of contrast enhancement. Only the low-energy and recombined images were displayed for review by radiologists. The CEM images would then be immediately reviewed by the session radiologist, and additional views (e.g., compression or magnified view) could be obtained if needed. The time required for each CEM examination was recorded, starting at contrast injection and concluding at the last image obtained.

Image interpretation

CEM images were retrospectively interpreted by two independent fellow radiologists (with more than eight years and two years of experience in breast imaging, respectively). Both radiologists were blinded to the clinical and histopathological information of the patients. In cases of discrepancy, the case was discussed, and a joint consensus was reached. Both low-energy and recombined images of CEM were reviewed. In cases with multicentric or multifocal lesions, the largest lesion was interpreted. The CEM imaging features were interpreted using the American College of Radiology Breast Imaging Reporting and Data System (ACR BI-RADS) lexicon for CEM, published in 2022. The imaging features analyzed include the presence of microcalcifications or architectural distortion on low-energy images and non-mass enhancement (NME) on recombined images. For low-energy images with associated enhancement on recombined images, shape and margins of mass, internal enhancement pattern, extent of enhancement, and lesion conspicuity were analyzed (Table 1).

**Table 1:** Imaging feature characteristics NME: non-mass enhancement

Imaging features	Findings
Low energy images	
Microcalcifications	Present or absent
Architectural distortion	Present or absent
Recombined images	
NME	Present or absent
Low-energy images with associated enhancement on recombined images	
Shape of mass	Oval/round/irregular
Margins of mass	Circumscribed/obscured/microlobulated/indistinct/spiculated
Internal enhancement pattern	Homogeneous/heterogeneous/rim
Extent of enhancement	Mammographic lesion partially enhances/completely enhances/enhancement extends beyond mammographic lesion/ no enhancement of the mammographic lesion but enhancement in the adjacent tissue
Lesion conspicuity	Low/moderate/high

Tissue sampling and analysis

Following surgery or an image-guided core biopsy, all the specimens were sent to the institute laboratory for immunohistochemistry analysis to detect the levels of ER, PR, and C-erb-B2 oncoprotein. Stained slides were evaluated by pathologists for nuclear ER or PR expression according to the American Society of Clinical Oncology/College of American Pathologists (ASCO/CAP) guidelines published in 2010 [9]: ≥1% cutoff for positive. According to the recommendation of the ASCO/CAP Clinical Practice Guideline Focused Update 2018 [10], C-erb-B2 oncoprotein on immunohistochemistry was based on the cell membrane staining pattern, with grade 0 or 1+ considered negative, 2+ considered equivocal, and grade 3+ considered positive. For equivocal specimens, further analysis with fluorescence in situ hybridization (FISH) was required. If the FISH ratio is higher than 2.2 or a HER2 gene copy is greater than 6.0, it is considered positive.

According to the St. Gallen Consensus Conference classification [11], breast cancer was categorized into luminal A, luminal B, HER2-enriched, and basal-like based on ER, PR, and HER2 expression status. The four molecular subtypes include (i) luminal A: ER-positive, PR-positive or PR-negative, and HER2-negative; (ii) luminal B: ER-positive, PR-positive or PR-negative, and HER2-positive; (iii) HER2-enriched: ER-negative and PR-negative and HER2-positive; and (iv) basal-like: ER-negative, PR-negative, and HER2-negative.

Statistical analysis

Statistical analysis was performed using SPSS Statistics (IBM Corp. IBM SPSS Statistics for Windows. Armonk, NY: IBM Corp.). The data was summarized using frequency and percentage for qualitative variables. The molecular subtypes were studied as dichotomous variables, including luminal, HER2, and basal-like. To assess the association between the CEM imaging features and molecular subtypes, contingency tables were used. The data was analyzed with a Fisher's exact test. Statistical significance was assumed when the p-value was <0.05.

## Results

This study consisted of 31 patients with 36 lesions. The mean age of patients was 52.67 years (median age: 53.50 years; range: 28-73 years). Out of the 36 lesions, 16 (44.4%) were luminal A, four (11.1%) were luminal B, 10 (27.8%) were HER2-enriched, and six (16.7%) were basal-like molecular subtypes. Thirty-five (97.2%) of the lesions showed the presence of a mass on recombined images, while one (2.8%) lesion showed the presence of NME only with no mass, which belonged to the HER2-enriched subtype.

**Figure 3:**
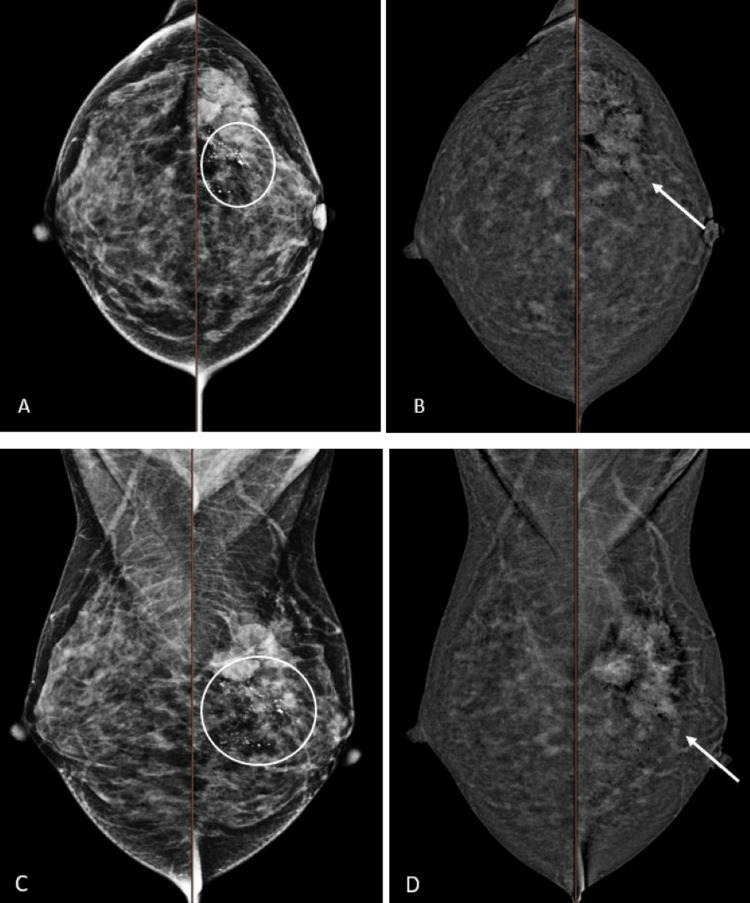
A 33-year-old female patient with histologically proven left breast HER2-enriched cancer. An irregular spiculated lesion in the left breast upper outer quadrant with heterogeneous enhancement (arrows). The mammographic lesion partially enhances with moderate lesion conspicuity. Associated fine pleomorphic microcalcifications with segmental distribution over the retroareolar to upper outer quadrant of the left breast (circles). A: CEM low energy, B: CEM recombined image of breasts in CC view, C: CEM low energy, D: CEM recombined image of breasts in MLO view CEM: contrast-enhanced mammography, CC: craniocaudal, MLO: mediolateral oblique, HER2: human epidermal growth factor receptor 2

**Figure 4:**
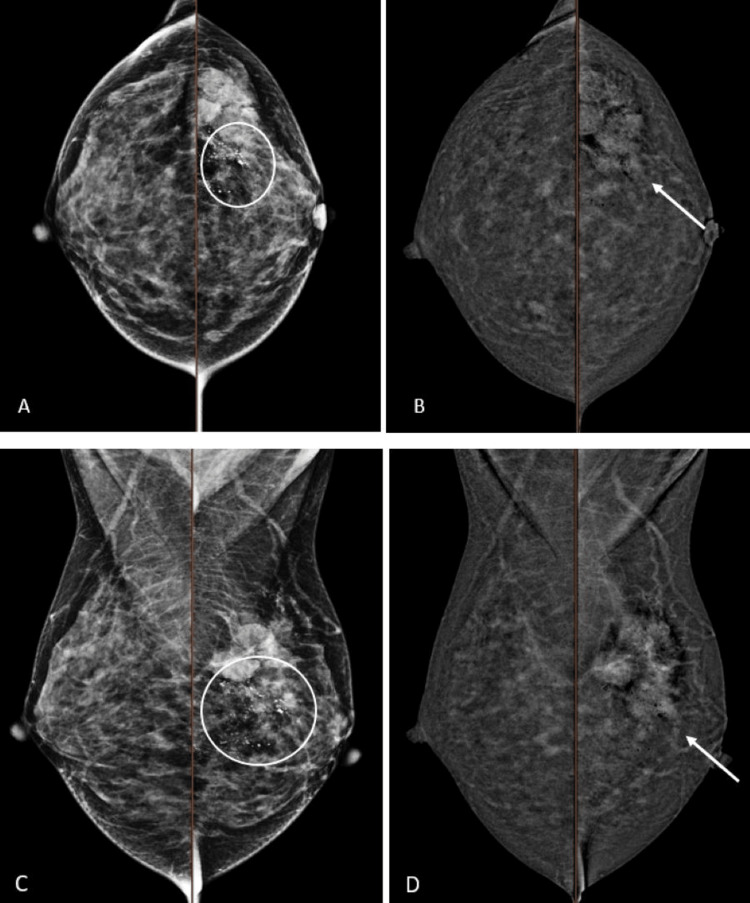
A 33-year-old female patient with histologically proven left breast HER2-enriched cancer. An irregular spiculated lesion in the left breast upper outer quadrant with heterogeneous enhancement (arrows). The mammographic lesion partially enhances with moderate lesion conspicuity. Associated fine pleomorphic microcalcifications with segmental distribution over the retroareolar to upper outer quadrant of the left breast (circles). A: CEM low energy, B: CEM recombined image of breasts in CC view, C: CEM low energy, D: CEM recombined image of breasts in MLO view CEM: contrast-enhanced mammography, CC: craniocaudal, MLO: mediolateral oblique, HER2: human epidermal growth factor receptor 2

**Figure 5:**
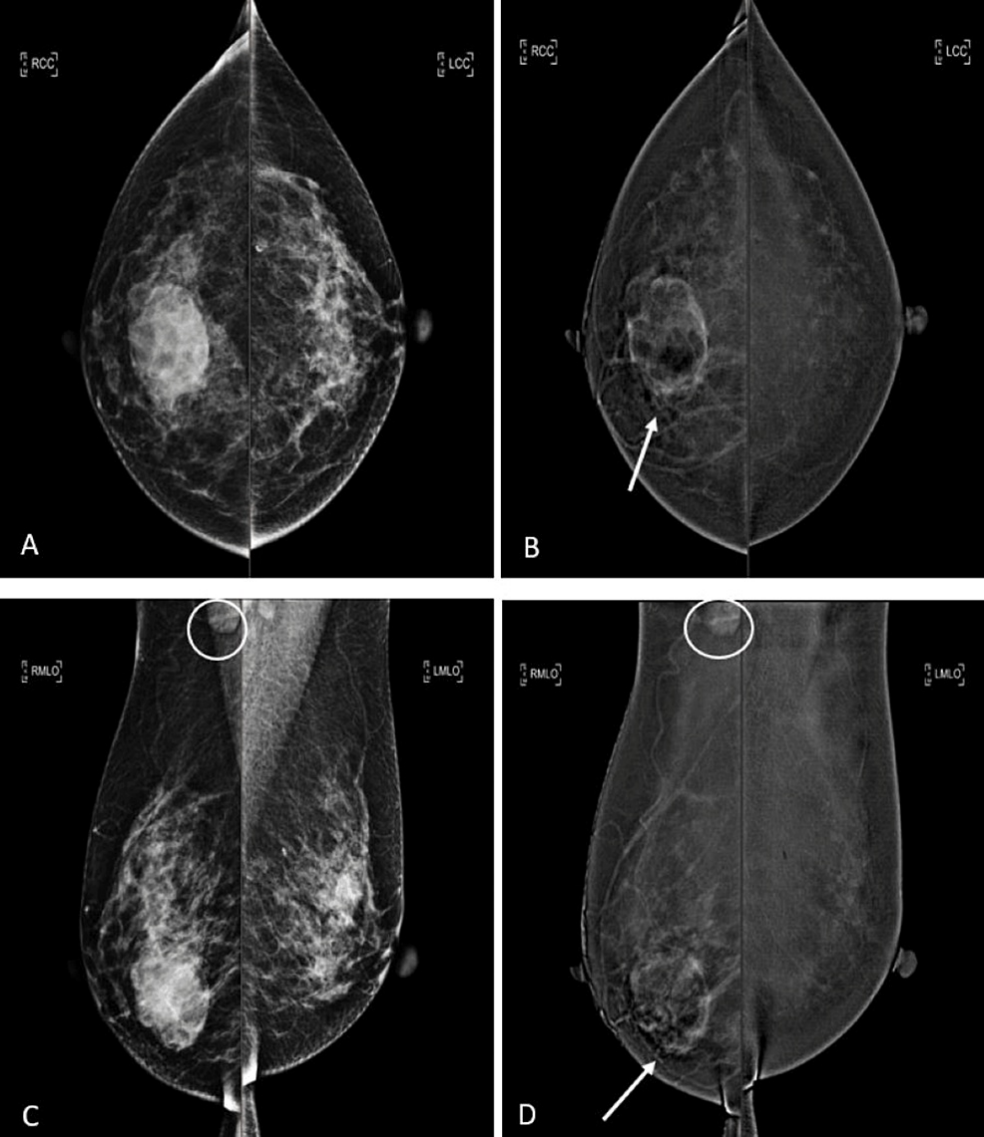
A 52-year-old female patient with histologically proven right breast basal-like cancer. An ovoid circumscribed lesion with rim enhancement at the right breast central lower portion (arrows). The mammographic lesion partially enhances with high lesion conspicuity. Prominent right axillary lymph node (circles). A: CEM low energy, B: CEM recombined image of breasts in CC view, C: CEM low energy, D: CEM recombined image of breasts in MLO view CEM: contrast-enhanced mammography, CC: craniocaudal, MLO: mediolateral oblique, HER2: human epidermal growth factor receptor 2

The correlation between CEM imaging findings and different breast cancer subtypes is summarized in Table 2. The presence of microcalcifications on low-energy images was the most common in the HER2-enriched subtype (90%; Figure 3), which showed a significant association (p=0.024, Table 2). On low-energy images with associated enhancement on recombined images, rim enhancement was the most common in the basal-like subtype (66.7%; Figure 4), which also showed a significant association (p=0.001, Table 2). The heterogeneous internal enhancement pattern was the most common in the non-basal-like subtype (82.8%; Figure 5), with a significant association (p=0.027, Table 2).

**Table 2:** Correlation between imaging findings and breast cancer subtypes A: Mammographic lesion partially enhances, B: mammographic lesion completely enhances, C: enhancement extends beyond the mammographic lesion, D: no enhancement of the mammographic lesion but enhancement in the adjacent tissue NME: non-mass enhancement

Imaging features	Luminal A	Non-luminal A	p-value	Luminal B	Non-luminal B	p-value	HER2-enriched	Non-HER2-enriched	p-value	Basal-like	Non-basal-like	p-value
Microcalcifications			0.544			0.185			0.024			0.21
Present	9/16 (56.3%)	12/20 (60%)		1/4 (25%)	20/32 (62.5%)		9/10 (90%)	12/26 (46.2%)		2/6 (33.3%)	19/30 (63.3%)	
Absent	7/16 (43.7%)	8/20 (40%)		3/4 (75%)	12/32 (37.5%)		1/10 (10%)	14/26 (53.8%)		4/6 (66.7%)	11/30 (36.7%)	
Architectural distortion			0.221			0.61			1			1
Present	3/16 (18.8%)	1/20 (5%)		0/4 (0%)	4/32 (12.5%)		1/10 (10%)	23/26 (88.5%)		0/6 (0%)	4/30 (13.3%)	
Absent	13/16 (81.2%)	19/20 (95%)		4/4 (100%)	28/32 (87.5%)		9/10 (90%)	3/26 (11.5%)		6/6 (100%)	26/30 (86.7%)	
NME			0.481			0.254			0.413			0.655
Present	5/16 (31.3%)	5/20 (25%)		0/4 (0%)	10/32 (31.3%)		4/10 (40%)	6/26 (23.1%)		1/6 (16.7%)	9/30 (30%)	
Absent	11/16 (68.7%)	15/20 (75%)		4/4 (100%)	22/32 (68.7%)		6/10 (60%)	20/26 (76.9%)		5/6 (83.3%)	21/30 (70%)	
Shape of mass												
Round	2/16 (12.5%)	2/19 (10.5%)	0.63	1/4 (25%)	3/31 (9.7%)	0.399	0/9 (0%)	4/26 (15.4%)	0.553	1/6 (16.7%)	3/29 (10.3%)	0.546
Oval	1/16 (6.3%)	4/19 (21.1%)	0.227	0/4 (0%)	5/31 (16.1%)	0.523	3/9 (33.3%)	2/26 (7.7%)	0.095	1/6 (16.7%)	4/29 (13.8%)	1
Irregular	13/16 (81.2%)	13/19 (68.4%)	0.319	3/4 (75%)	23/31 (74.2%)	0.732	6/9 (66.7%)	20/26 (76.9%)	0.665	4/6 (66.7%)	22/29 (75.9%)	0.635
Margin of mass												
Circumscribed	3/16 (18.8%)	4/19 (21.1%)	0.602	0/4 (0%)	7/31 (22.6%)	0.391	2/9 (22.2%)	5/26 (19.2%)	1	2/6 (33.3%)	5/29 (17.2%)	0.576
Obscured	2/16 (12.5%)	4/19 (21.1%)	0.418	2/4 (50%)	4/31 (12.9%)	0.128	1/9 (11.1%)	5/26 (19.2%)	1	1/6 (16.7%)	5/29 (17.2%)	1
Microlobulated	0/16 (0%)	0/19 (0%)	-	0/4 (0%)	0/31 (0%)	-	0/9 (0%)	0/26 (0%)	-	0/6 (0%)	0/29 (0%)	-
Indistinct	3/16 (18.8%)	6/19 (31.6%)	0.319	0/4 (0%)	9/31 (29%)	0.286	3/9 (33.3%)	6/26 (23.1%)	0.665	3/6 (50%)	6/29 (20.7%)	0.162
Spiculated	8/16 (50%)	5/19 (26.3%)	0.137	2/4 (50%)	11/31(35.5%)	0.478	3/9 (33.3%)	10/26 (38.5%)	1	0/6 (0%)	13/29 (44.8%)	0.064
Internal enhancement pattern												
Homogeneous	3/16 (37.5%)	1/19 (5.3%)	0.238	1/4 (25%)	3/31 (9.7%)	0.399	0/9 (0%)	4/26 (15.4%)	0.553	0/6 (0%)	4/29 (13.8%)	1
Heterogeneous	13/16 (81.2%)	13/19 (68.4%)	0.319	3/4 (75%)	23/31 (74.2%)	0.732	8/9 (88.9%)	18/26 (69.2%)	0.391	2/6 (33.3%)	24/29 (82.8%)	0.027
Rim	0/16 (0%)	5/19 (26.3%)	0.036	0/4 (0%)	5/31 (16.1%)	0.523	1/9 (11.1%)	4/26 (15.4%)	1	4/6 (66.7%)	1/29 (11.1%)	0.001
Extent of enhancement												
A*	6/16 (35%)	9/19 (47.4%)	0.404	1/4 (25%)	14/31 (45.2%)	0.419	4/9 (44.4%)	11/26 (42.3%)	1	4/6 (66.7%)	11/29 (37.9%)	0.367
B*	8/16 (50%)	7/19 (36.8%)	0.33	3/4 (75%)	12/31 (38.7%)	0.2	2/9 (22.2%)	13/26 (50%)	0.244	2/6 (33.3%)	13/29 (44.8%)	0.68
C*	2/16 (12.5%)	3/19 (15.8%)	0.585	0/4 (0%)	5/31 (16.1%)	0.523	3/9 (33.3%)	2/26 (7.7%)	0.095	0/6 (0%)	5/29 (17.2%)	0.561
D*	0/16 (0%)	0/19 (0%)	-	0/4 (0%)	0/31 (0%)	-	0/9 (0%)	0/26 (0%)	-	0/6 (0%)	0/29 (0%)	-
Lesion conspicuity												
Low	3/26 (18.8%)	2/19 (10.5%)	0.415	0/4 (0%)	5/31 (16.1%)	0.523	2/9 (22.2%)	3/26 (11.5%)	0.586	0/6 (0%)	5/29 (17.2%)	0.561
Moderate	3/16 (18.8%)	7/19 (36.8%)	0.212	2/4 (50%)	8/31 (25.8%)	0.319	3/9 (33.3%)	7/26 (26.9%)	0.694	2/6 (33.3%)	8/29 (27.6%)	1
High	10/16 (62.5%)	10/19 (52.6%)	0.404	2/4 (50%)	18/31 (58.1%)	0.581	4/9 (44.4%)	16/26 (61.5%)	0.451	4/6 (66.7%)	16/29 (55.2%)	0.68

On low-energy images, the presence of architectural distortion was most common in the luminal A subtype (18.8%). There was no statistically significant correlation between the presence of architectural distortion and different molecular subtypes (p=0.221, p=0.610, p=1.000, and p=1.000 for luminal A, luminal B, HER2-enriched, and basal-like subtypes, respectively).

The presence of NME on recombined images was the most common in the HER2-enriched subtype (40%), with no statistically significant association (p=0.413). There was also no statistically significant association between NME and luminal A (p=0.481), luminal B (p=0.254), and basal-like subtype (p=0.655).

The association between molecular subtypes and various imaging features on low-energy images with associated enhancement on recombined images was analyzed. Irregular was the most common shape among the subtypes, but there was no statistically significant association noted (luminal A: 81.2%, p=0.319; luminal B: 75%, p=0.732; HER2-enriched: 66.7%, p=0.665; basal-like: 66.7%, p=0.635). Homogeneous internal enhancement patterns were the most common in the luminal A subtype (37.5%), with no statistically significant correlation noted (p=0.238). There was no statistically significant association between the extent of enhancement as well as lesion conspicuity and the molecular subtype (all p>0.05).

## Discussion

Breast cancer is a diverse disease with distinct molecular subtypes that have significant therapeutic and prognostic values. Molecular subtyping is an essential therapeutic requirement nowadays for customized treatments, while breast imaging is one of the most important components in disease diagnosis. A reliable correlation between imaging features and molecular subtypes can help in improving patient care through prompt diagnosis [12].

CEM is an emerging breast imaging modality and a functional imaging method that demonstrates the vascularity and neo-angiogenesis of breast lesions using intravenous contrast and dual-energy subtracted images, which has increased usage in clinical practice in recent years [13]. With the introduction of the ACR BI-RADS lexicon for CEM, CEM reporting has become more structured and standardized. The ACR BI-RADS lexicon includes a description of lesion conspicuity and extent of enhancement, which are new descriptors for breast imaging. Published studies have shown that CEM has similar accuracy to MRI for the detection of breast cancer [14] and is often better tolerated by patients [15].

In this study, we evaluated the association between CEM imaging features and molecular subtypes of breast cancer. The imaging features were analyzed according to the ACR BI-RADS lexicon. The results showed that three features-the presence of microcalcifications, heterogeneous, and rim internal enhancement patterns-were correlated with molecular subtypes.

The presence of microcalcifications on low-energy CEM images is associated with the HER2-enriched subtype (p=0.024). Several previous studies have shown the presence of microcalcifications on mammograms is associated with the HER2-enriched subtype [16-18]. Low-energy images of CEM are obtained using energy below the k-edge of iodine, with contrast materials not depicted. The low-energy image appears similar to a full-field digital mammography (FFDM). Some studies have shown that the image quality of low-energy CEM is similar or even better when compared to FFDM and is diagnostically equivalent to FFDM despite the presence of intravenous contrast [19-20]. Compared to FFDM, CEM has better sensitivity and specificity for breast cancer detection [21-22]. The ability to evaluate microcalcifications is also an advantage of CEM over MRI. Our study has shown that the presence of microcalcifications on low-energy CEM images, like in mammograms, is associated with the HER2-enriched subtype. Calcifications are usually considered a marker for intraductal compartments associated with invasive breast cancer, with the dominant pathological process being necrosis [23].

CEM, apart from demonstrating the anatomic changes of the breast, also shows the local changes in breast perfusion caused by tumor angiogenesis. Unlike MRI, enhancement kinetic curves are not used with CEM because there is only a single time point for each image. CEM has its own enhancement characteristics described by the ACR BI-RADS lexicon for CEM. In this study, rim enhancement on recombined images is associated with a basal-like subtype (p=0.001), and heterogeneous enhancement is associated with a non-basal-like subtype (p=0.027). Some previous studies have shown that rim enhancement on MRI is associated with basal-like subtype cancer [24-25], and this study shows that rim enhancement on CEM also has the same association. The rim enhancement of basal-like tumors may be attributed to their high growth rate, local invasiveness, and internal necrosis [26].

Most of the invasive breast cancer included in this study was luminal A (44.4%), while luminal B (11.1%), HER2-enriched (27.8%), and basal-like (16.7%) subtypes were the minorities. This reflects the lower frequency of luminal B, HER2-enriched, and basal-like subtypes and is compatible with some previous studies [27].

According to the ACR BI-RADS lexicon, NME is described with different distributions and internal enhancement patterns. However, due to the small number of patients in our study with NME of a particular distribution or internal enhancement, such an association could not be statistically examined. Only the presence or absence of NME was included in our study. Further research is required to evaluate the association of NME distribution and internal enhancement patterns with molecular subtypes.

Previous studies have shown that multicentric or multifocal disease detected on MRI is more common in the HER2-enriched subtype [28]. In our study, the number of patients with multicentric or multifocal disease of each molecular subtype was small (luminal A: 3, luminal B: 1, HER2-enriched: 4, basal-like: 3), thus the association could not be statistically evaluated. The association of multicentric or multifocal disease detected on CEM with molecular subtypes requires further research.

The study had some limitations. First, it was a single institution study with a small number of patients and only one mammogram machine with CEM. Therefore, the sample size was small, and the number of luminal B, HER2-enriched, and basal-like subtypes was even smaller. More multi-institution studies with larger sample sizes are warranted in the future. Second, selection bias may have been present due to the retrospective nature of the study. Third, patients who had breast cancer treatment prior to CEM were excluded; the results may not be applicable to locally advanced breast cancer or patients with a past history of breast cancer. Lastly, the CEM images were evaluated by two readers in consensus, and the inter-observer variability in image interpretation was not considered. It is worth noting that the possibility of intratumoral heterogeneity may contribute to discordance of ER, PR, and HER2 status upon histopathological assessment on core biopsy and thus possible implications on breast cancer subtyping.

## Conclusions

CEM is an emerging breast imaging tool that demonstrates both the anatomy and neovascularity of breasts. CEM imaging features, including the presence of calcifications and certain internal enhancement patterns, were correlated to distinguishing breast cancer molecular subtypes. Although histopathology remains the standard of care, CEM has the potential to distinguish different subtypes of breast cancer. CEM can thus be a useful, non-invasive tool for management planning and prognosis evaluation.
